# Supplementary material for: Genetic Diversity of Norovirus in Children with Acute Gastroenteritis in Southwest Nigeria, 2015–2017
Source: Viruses. 2023 Feb 28;15(3):644. doi: 10.3390/v15030644 (PMC10056664; doi:10.3390/v15030644)
Supplement: Supplementary file 1 [file viruses-15-00644-s001.zip › viruses-2242354-supplementary.pdf]

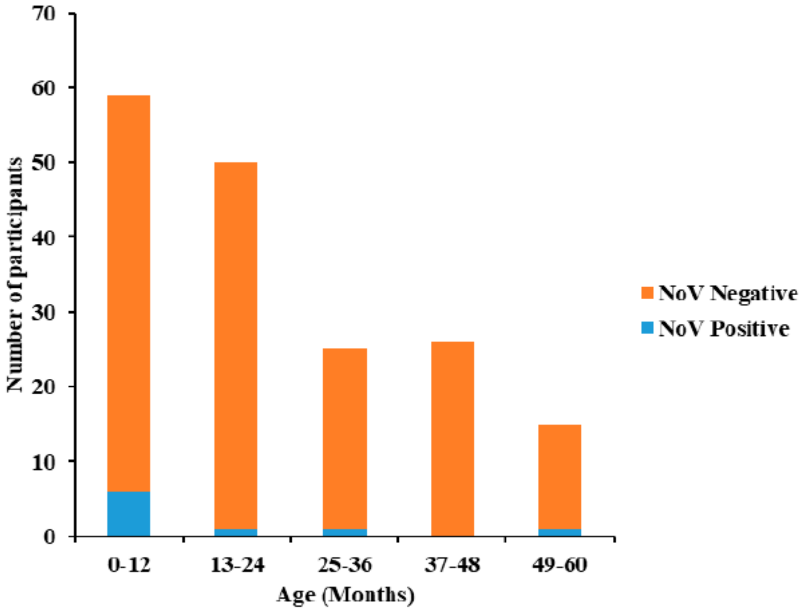

Figure S1. Age distribution of NoV infection in children with acute gastroenteritis in Ogun State, Nigeria, from February 2015 to April 2017.
